# *Clonorchis sinensis* MF6p/HDM (CsMF6p/HDM) induces pro-inflammatory immune response in RAW 264.7 macrophage cells *via* NF-κB-dependent MAPK pathways

**DOI:** 10.1186/s13071-020-3882-0

**Published:** 2020-01-13

**Authors:** Jung-Mi Kang, Won Gi Yoo, Hương Giang Lê, Jinyoung Lee, Woon-Mok Sohn, Byoung-Kuk Na

**Affiliations:** 1 Department of Parasitology and Tropical Medicine, and Institute of Health Sciences, Gyeongsang National University College of Medicine, Jinju, 52727 Republic of Korea; 2 BK21Plus Team for Anti-aging Biotechnology and Industry, Department of Convergence Medical Science, Gyeongsang National University, Jinju, 52727 Republic of Korea; 3 Department of Medical Environmental Biology, Chung-Ang University College of Medicine, Seoul, 06974 Republic of Korea; 4 Present Address: Department of Tropical Medicine, and Inha Research Institute for Medical Sciences, Inha University College of Medicine, Incheon, 22212 Republic of Korea

**Keywords:** *Clonorchis sinensis*, MF6p/host defense molecule, Lipopolysaccharide, Pro-inflammatory immune response, Structure, Docking

## Abstract

**Background:**

MF6p/host defense molecules (HDMs) are a broad family of small proteins secreted by helminth parasites. Although the physiological role of MF6p/HDMs in trematode parasites is not fully understood, their potential biological function in maintaining heme homeostasis and modulating host immune response has been proposed.

**Methods:**

A gene encoding the MF6p/HDM of *Clonorchis sinensis* (CsMF6p/HDM) was cloned. Recombinant CsMF6p/HDM (rCsMF6p/HDM) was expressed in *Escherichia coli*. The biochemical and immunological properties of rCsMF6/HDM were analyzed. CsMF6p/HDM induced pro-inflammatory response in RAW 264.7 cells was analyzed by cytokine array assay, reverse transcription polymerase chain reaction, and enzyme-linked immunosorbent assay. The structural feature of CsMF6p/HDM was analyzed by three-dimensional modeling and molecular docking simulations.

**Results:**

The CsMF6p/HDM shares a high level of amino acid sequence similarity with orthologs from other trematodes and is expressed in diverse developmental stages of the parasite. The rCsMF6p/HDM bound to bacteria-derived lipopolysaccharide (LPS), without effectively neutralizing LPS-induced inflammatory response in RAW 264.7 macrophage cells. Rather, the rCsMF6p/HDM induced pro-inflammatory immune response, which is characterized by the expression of TNF-α and IL-6, in RAW 264.7 cells. The rCsMF6p/HDM-induced pro-inflammatory immune response was regulated by JNK and p38 MAPKs, and was effectively down-regulated *via* inhibition of NF-κB. The structural analysis of CsMF6p/HDM and the docking simulation with LPS suggested insufficient capture of LPS by CsMF6p/HDM, which suggested that rCsMF6p/HDM could not effectively neutralize LPS-induced inflammatory response in RAW 264.7 cells.

**Conclusions:**

Although rCsMF6p/HDM binds to LPS, the binding affinity may not be sufficient to maintain a stable complex of rCsMF6p/HDM and LPS. Moreover, the rCsMF6p/HDM-induced pro-inflammatory response is characterized by the release of IL-6 and TNF-α in RAW 264.7 macrophage cells. The pro-inflammatory response induced by rCsMF6p/HDM is mediated *via* NF-κB-dependent MAPK signaling pathway. These results collectively suggest that CsMF6p/HDM mediates *C. sinensis*-induced inflammation cascades that eventually lead to hepatobiliary diseases.
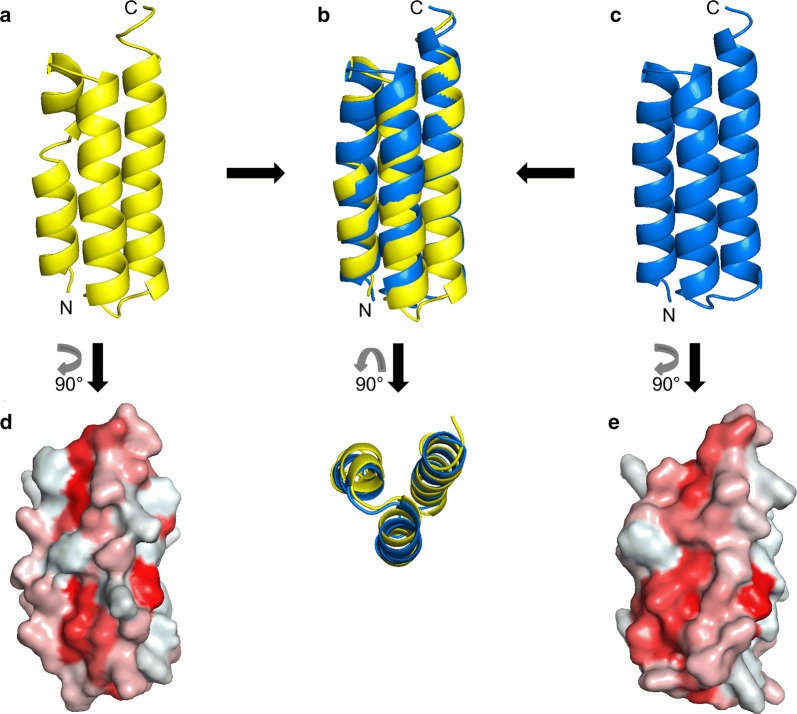

## Background

Clonorchiasis is a zoonotic food-borne parasitic disease with a serious impact on public health [1]. It has been estimated that more than 15 million people worldwide suffer from clonorchiasis and over 200 million are at risk of infection [2]. The disease is caused by a species of carcinogenic liver fluke, *Clonorchis sinensis*, which is widely distributed in Korea, China and northern Vietnam [1, 3, 4]. Humans are usually infected with the parasite by eating raw or undercooked freshwater fish containing metacercariae. Inside the bile ducts, the parasite induces mechanical damage to biliary epithelium, and generates an inflammatory responses and diverse clinical manifestations such as epithelial hyperplasia, periductal fibrosis and cholangiocarcinoma [5]. Thus, *C. sinensis* has been regarded as a group I biological carcinogen by the International Agency for Research on Cancer of the World Health Organization [6].

The MF6p/host defense molecule (HDM) of *C. sinensis* (CsMF6p/HDM) is a small protein consisting of 90 amino acids and was initially reported as a 7 kDa antigen of unknown function [7, 8]. However, it has recently been classified under a new family of heme-binding proteins (MF6p/HDMs) with homologues from the other flukes including *Fasciola hepatica* (FhMF6p/HDM), *Opisthorchis viverrini* (OvMF6p/HDM) and *Paragonimus westermani* (PwMF6p/HDM) [9]. The physiological role of MF6p/HDMs in trematode parasites has yet to be elucidated. However, several studies investigating FhMF6p/HDM suggested a role in host immune modulation [10–13]. Moreover, the increased interest in this molecule has been attributed to its heme-binding ability, and its role as a heme scavenger and transporter to maintain heme homeostasis in trematode parasites [9, 14]. The heme-scavenging ability is essential for the survival of blood-feeding trematodes since large amount of heme, which is toxic as a free form, are released from the catabolism of host erythrocytes [15]. These findings collectively suggest that trematode MF6p/HDMs are attractive targets for the development of vaccine and therapeutic drugs.

In this study, the biochemical and immune modulation properties of CsMF6p/HDM were characterized. The recombinant CsMF6p/HDM (rCsMF6p/HDM) bound to lipopolysaccharide (LPS) but did not effectively neutralize LPS-induced pro-inflammatory responses in RAW 264.7 macrophage cells. Further, rCsMF6p/HDM induced pro-inflammatory immune responses in RAW 264.7 macrophage cells *via* NF-κB-dependent MAPK pathways.

## Methods

### Parasites and sera

Metacercariae of *Clonorchis sinensis* were collected from the naturally infected intermediate host, *Pseudorasbora parva*, collected in Korea. Sprague-Dawley rats were experimentally infected with 100 metacercariae by oral feeding. The rats were sacrificed at 2, 4, 6 and 9 weeks post-infection [16]. Juvenile and adult worms were collected from the livers and washed five times with cold phosphate-buffered saline (PBS, pH 7.4) to remove any host contamination. The collected parasites were stored at − 70 °C until use, or used directly for RNA preparation to synthesize cDNA. The sera of infected rats were also collected at the time of sacrifice [16].

### Expression and purification of recombinant CsMF6p/HDM (rCsMF6p/HDM)

The gene encoding CsMF6p/HDM (GenBank: AF281362.1) [7, 8] was amplified by polymerase chain reaction (PCR) from the cDNA of adult *C. sinensis* and cloned into T&A cloning vector (Real Biotech Corporation, Banqiao City, Taiwan). The nucleotide sequence of the cloned CsMF6p/HDM gene was confirmed by sequencing. To produce the recombinant CsMF6p/HDM (rCsMF6p/HDM), a partial CsMF6p/HDM without the N-terminal signal peptide region was amplified using the following primers; forward (5′-GGA TCC CGT CCC AGT GAG GAG ACC CGT-3′) and reverse (5′-AAG CTT TCA CTC CCC AAC GTA AGT CTC-3′) (restriction sites underlined). The purified PCR product was ligated into the T&A cloning vector (Real Biotech Corporation) and the ligate was transformed into *Escherichia coli* DH5α. The resulting plasmid DNA was digested with *Bam*HI and *Hind*III, ligated into the pQE-9 expression vector (Qiagen, Hilden, Germany), and then transformed into *E. coli* M15 [pREP4] cells (Qiagen). Selected clone was grown and induced with 1 mM isopropyl-1-thio-β-d-galactopyranoside (IPTG). The bacteria were suspended in native lysis buffer (50 mM NaH_2_PO_4_, 300 mM NaCl, 10 mM imidazole, pH 8.0), sonicated on ice and centrifuged at 4 °C for 20 min at 12,000× *rpm*. The rCsMF6p/HDM was purified from the supernatant by nickel-nitrilotriacetic acid (Ni-NTA) resin (Qiagen) according to the manufacturer’s protocols. The purification and purity of the rCsMF6p/HDM was confirmed by 15% sodium dodecyl sulfate-polyacrylamide gel electrophoresis (SDS-PAGE).

### Production of polyclonal antibody for rCsMF6p/HDM (anti-CsMF6p/HDM)

The anti-CsMF6p/HDM was produced by immunizing BALB/c mice with the purified rCsMF6p/HDM. The protein (100 μg) was mixed with Freund’s complete or incomplete adjuvant (Sigma-Aldrich, St. Louis, MO, USA) and injected into mice intraperitoneally three times at 2-week intervals. Two weeks after the final booster, the mice were sacrificed and the sera were collected. The immunoglobulin G (IgG) fraction was further purified with the Protein G agarose (Pierce, Rockford, lL, USA) according to the manufacturer’s instructions. The specificity of the anti-CsMF6p/HDM was confirmed by immunoblot analysis.

### Expression profile of CsMF6p/HDM in different developmental stages

The expression level of CsMF6p/HDM was investigated *via* semi-quantitative reverse transcription PCR (RT-PCR) and immunoblot analysis across different developmental stages of *C. sinensis*; metacercariae, 2-week-old juveniles, and 4-, 6- and 9-week-old adults. The total RNA was extracted from each developmental stage of worms using RNAiso (Takara, Ostu, Japan) according to the manufacturer’s instructions. The total RNA was quantified by spectrophotometry and equalized, and its purity was verified on a 1% agarose gel. Reverse transcription and subsequent amplification of CsMF6p/HDM transcripts were performed with the same amounts of total RNA (1 μg each) and specific primers using the ProtoScript® II RT-PCR Kit (Invitrogen, Carlsbad, CA, USA) according to the manufacturer’s instruction. PCR was performed using a primer set specific for CsMF6p/HDM (forward: 5′-CGT CCC AGT GAG GAG ACC CGT-3′, reverse: 5′-CAC TCC CCA ACG TAA GTC TC-3′). As an internal control, the *C. sinensis* actin (Cs actin) gene (GenBank: EU109284) was used as previously described [16]. The amplicons were analyzed on a 2% agarose gel, visualized with RedSafe^TM^ nucleic acid staining solution (iNtRON Biotechnology Inc., Seongnam, Korea) and observed under ultraviolet light. For immunoblot, worms of each developmental stage of *C. sinensis* were homogenized in PBS containing the Complete^TM^ protease inhibitor cocktails (Roche, Mannheim, Germany) and the supernatants were collected. The protein concentration in each worm extract was measured with Quick Start™ Bradford Protein Assay kit (Bio-Rad, Hercules, CA, USA) according to the manufacturer’s instructions. The same amount of worm extract (10 μg) from each developmental stage was separated on 15% SDS-PAGE and transferred to nitrocellulose membrane (Bio-Rad). The membrane was blocked with PBS supplemented with 0.05% Tween 20 (PBST, pH 7.4) and 3% skimmed milk for 1 h at room temperature. The membrane was then incubated with anti-CsMF6p/HDM diluted 1:1000 in PBST at room temperature for 2 h. After several washes with PBST, the membrane was incubated with 1:1000 diluted horseradish peroxidase (HRP)-conjugated anti-mouse IgG (Sigma-Aldrich). The immuno-reactive bands were visualized with 4-chloro-1-naphthol (Sigma-Aldrich) and the reaction was terminated by washing the membrane with distilled water.

### Lipopolysaccharide (LPS) binding assay

A dot blot was used to evaluate the binding profile of rCsMF6p/HDM with LPS. Nitrocellulose membrane (Bio-Rad, pore size: 0.25 μm) was immersed in PBS for 10 min and fixed into a Bio-Dot microfiltration apparatus (Bio-Rad). LPS (*E. coli* strain O127:B8, Sigma-Aldrich) dissolved in PBS (10 μg/100 μl) was added to the wells of the apparatus and the sample was allowed to pass through the membrane under gravity. Serial dilutions of rCsMF6p/HDM (0 to 20 μg) in PBS were applied to each well and incubated for 1 h at room temperature. After incubation, the membrane was removed from the apparatus and blocked with PBST containing 3% skimmed milk for 1 h at room temperature. The membrane was incubated with anti-CsMF6p/HDM diluted 1:1000 in PBST at room temperature for 2 h. After several washes with PBST, the membrane was incubated with 1:1000 diluted HRP-conjugated anti-mouse IgG (Sigma-Aldrich). The immuno-reactive dots were visualized with 4-chloro-1-naphthol (Sigma-Aldrich) and the reaction was terminated by washing the membrane with distilled water.

### Cultivation of RAW 264.7 cells and treatment with rCsMF6p/HDM

RAW 264.7 murine macrophage cells (ATCC^®^ TIB-71^TM^) were purchased from American Type Culture Collection (Manassas, VA, USA) and maintained at 37 °C with 5% CO_2_ in Dulbecco’s modified Eagle’s medium (DMEM; Welgene Inc., Daegu, Korea) containing 10% heat-inactivated fetal bovine serum (FBS; Gibco, Grand Island, NY, USA), 1% l-glutamine (Welgene Inc.) and 1% penicillin/streptomycin (Gibco). To evaluate the potential cytotoxic effect of rCsMF6p/HDM to RAW 264.7 cells, the cell viability of RAW 264.7 cells after treatment with rCsMF6p/HDM was assessed using the methyl thiazolyl tetrazolium (MTT) method. To remove any LPS contamination in purified rCsMF6p/HDM, the Detoxi-gel endotoxin removing column (Pierce) was used according to the manufacturer’s instruction. No residual amount of endotoxin was detected in the sample when analyzed with Pierce™ LAL Chromogenic Endotoxin Quantitation Kit (Pierce). The LPS-depleted rCsMF6p/HDM was filtered with a syringe filter (0.22 μm; Millipore, Billerica, MA, USA) and used for further experiments. RAW 264.7 cells were continuously deprived of serum by incubation in 1% FBS overnight, followed by incubation in serum-free medium for 12 h. The serum-starved cells were seeded in a 96-well microplate (2 × 10^4^ cells/well) and incubated for 6 h. The cells were treated with different concentrations of rCsMF6p/HDM (0 to 50 μg/ml) and incubated at 37 °C for 24 h. The supernatant was then drained from the plate and 100 μl of MTT (3-(4,5-Dimethylthiazol-2-yl)-2,5-Diphenyltetrazolium Bromide) (2 mg/ml; Sigma-Aldrich) were added in each well. The plate was further incubated for 3 h at 37 °C then followed by the addition of 100 μl of dimethyl sulfoxide (DMSO; Sigma-Aldrich) to each well. The plate was incubated for 30 min at 37 °C and the reaction was read at 595 nm with a Multiskan FC microplate reader (Thermo, Vantaa, Finland). All the experiments were performed in triplicate, and the mean and standard deviation (SD) were calculated. No cellular damage was observed when cells were treated with 20 μg/ml or less of rCsMF6p/HDM.

### Cytokine array assay

To analyze the immune response of RAW 264.7 cells induced by rCsMF6p/HDM, the cells were seeded at ~ 70% confluence on a 6-well plate and grown for 24 h under standard culture conditions as described above. Cells were continuously deprived of serum as described above followed by the addition of rCsMF6p/HDM (10 μg/ml) and incubation for 12 h. For negative control, PBS-treated RAW 264.7 cells were used. The expression profiles of cytokines/chemokines in RAW 264.7 cells, which was stimulated by rCsMF6p/HDM, were analyzed compared with the control cells treated with PBS. The culture supernatant was harvested and centrifuged at 1200× *rpm* for 5 min at 4 °C. The supernatant was subsequently analyzed using the Proteome Profiler^TM^ Mouse Cytokine Array Panel A (R&D systems, Minneapolis, MN, USA) according to the manufacturer’s instructions.

### LPS neutralization assay

To determine the ability of rCsMF6p/HDM to neutralize or modulate LPS-induced inflammatory immune response in RAW 264.7 cells, different concentrations of rCsMF6p/HDM (5 or 10 μg) were incubated with LPS (1 μg) in PBS for 2 h at room temperature. Each mixture was added to serum-starved RAW 264.7 cells cultured in a 6-well plate and incubated for 12 h. The cells were harvested and the expression profiles of IL-6 and TNF-α were analyzed by RT-PCR. Total RNA was extracted from the cells by using RNAiso (Takara) according to the manufacturer’s instructions. RT-PCR was performed using the same method described above. PCR was carried out using specific primer sets for the mouse IL-6, TNF-α and GAPDH genes: IL-6, forward primer (5′-CCG GAG AGG AGA CTT CAC AG-3′) and reverse primer (5′-GGA AAT TGG GGT AGG AAG GA-3′); TNF-α, forward primer (5′-CAT CCT CTC AAA ATT CGA GTG ACA-3′) and reverse primer (5′-TGG GAG TAG ACA AGG TAC AAC CC-3′); and GAPDH, forward primer (5′-ACC AGA GTC CAT GCC ATC AC-3′) and reverse primer (5′-CAC CAC CCT GTT GCT GTA GCC-3′). The amplicon sizes were confirmed as described above and each band was quantified using ImageJ v1.44 software [17].

### Analysis of inflammatory immune responses in RAW 264.7 cells induced by rCsMF6p/HDM

To analyze the inflammatory response in RAW 264.7 cells induced by rCsMF6p/HDM, the effects of MAPK inhibitors on IL-6 and TNF-α production in the cells were analyzed. Inhibitors for p38 (SB203580), c-Jun N-terminal kinase (JNK) (SP600125), extracellular signal-regulated protein kinase (ERK) (U0126), NF-κB (MG132) and AP-1 (SR11302) were used in this study. All inhibitors were purchased from Calbiochem (San Diego, CA, USA). RAW 264.7 cells were seeded in 6-well dishes (2 × 10^5^ cells/well) and cultured to approximately 70% confluence. After changing the media with fresh serum-free media, different concentrations of each inhibitor were added to the cells and incubated for 3 h. Next, rCsMF6p/HDM (10 µg/ml) was added to the cells pretreated with each inhibitor followed by incubation for an additional 12 h. RAW 264.7 cells treated with the same volume of PBS and LPS (1 µg/ml) were used as negative and positive controls, respectively. Cells treated with only rCsMF6p/HDM (10 µg/ml) were also included as a control. The cells were harvested and washed with PBS. The total RNA was isolated using the same protocols described above followed by RT-PCR for IL-6 and TNF-α as described above. The relative levels of IL-6 and TNF-α in each cell supernatant were also quantified by enzyme-linked immunosorbent assay (ELISA) using the mouse Quantikine IL-6 and TNF-α ELISA kits (R&D systems, Minneapolis, MN, USA) according to the manufacturer’s instructions.

### Primary sequence similarity and features

Amino acid sequences of CsMF6p/HDM and homologues of *Fasciola hepatica* (FhMF6p/HDM), *Opisthorchis viverrini* (OvMF6p/HDM) and *Paragonimus westermani* (PwMF6p/HDM) were retrieved from the NCBI GenBank database [18]. The signal peptide region was predicted by SignalP v4.0 [19] and removed to develop a three-dimensional (3D) structure model. Multiple sequence alignments were generated using MAFFT v7.407 [20] and displayed by Jalview [21]. Protein physicochemical properties were predicted using the AA-Prop (http://www.biogem.org/tool/aa-prop).

### Threading-based modeling and analysis of 3D structure

To obtain the most accurate structures of MF6p/HDM homologues, the top five 3D models of CsMF6p/HDM, FhMF6p/HDM, OvMF6p/HDM and PwMF6p/HDM were predicted using I-TASSER (Iterative Threading ASSEmbly Refinement) [22]. The C-score, ranging from − 5 to 2, corresponds to a high confidence of the model. The structural similarity matrix was generated by measuring the pairwise structural similarities of 20 models based on the Dali Z-scores [23] using the Dali server [24]. The Dali Z-score higher than 2 indicates fold similarity. Based on the similarity matrix, a structural dendrogram was derived using average linkage clustering [24]. Both the backbone and the side chain of final 3D models were obtained by refinement using GalaxyRefine with the “both mild and aggressive relaxation” option [25]. The quality of the models was evaluated using Ramachandran plot [26], ERRAT [27] and ProSA [28]. A structural similarity heatmap image was generated with Plotly (https://plot.ly) and a structural similarity dendrogram was displayed using Evolview v2 [29]. The overall structural superposition was performed using the TM-align [30]. The TM-score value corresponds to the overall fold similarity. The TM-score ranges from 0 to 1, where 1 indicates 100% similarity between the two structures. Structure visualization was performed using PyMOL (http://www.pymol.org) and the hydrophobic surface was applied by running the python script “Color_h” (https://pymolwiki.org/index.php/Color_h).

### Molecular docking simulation

The PDB file format was corrected using PDBEditor [31]. LPS (Compound ID 11970143) was retrieved as sdf format from PubChem database [32] and the format was converted to mol2 format using Open Babel [33]. Molecular docking simulations of MF6p/HDM homologues and LPS were conducted using AutoDock Vina [34] in DockingServer [35]. A box size of 22 × 40 × 24 Å and box center of 51.6 × 51.5 × 51.3 for CsMF6p/HDM, and a box size of 27 × 34 × 31 Å and box center of 50.2 × 50.1 × 50.3 for FhMF6p/HDM were used for each simulation. Each docking simulation was performed using a setup of 99 different runs (‘ga_run’), 50,000,000 energy evaluations (‘ga_num_evals’) and 540,000 generations (‘ga_num_generation’) in order to terminate when either limit is reached. The remaining options were set as default values. A plot was generated using DataGraph v3.0 (Visual Data Tools Inc., Chapel Hill, NC, USA).

## Results

### Sequential conservation of trematode MF6p/HDM homologues

**Fig. 1:**
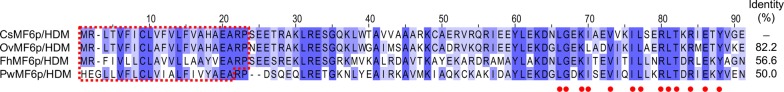
Sequence conservation of CsMF6p/HDM and other flukes’ homologues. The deduced amino acid sequences of CsMF6p/HDM (GenBank: AAM55183.1) was aligned with MF6p/HDMs of *O. viverrini* (GenBank: ES416124.1), *F. hepatica* (GenBank: CCA61804.1), *P. westermani* (GenBank: AT007125.1). The shading represented the degree of identity among the sequences, ranging blue (100%) to white (0%). Sequence identities were represented in the right. The signal peptide is indicated by the box with dotted red line. Closed circle in red indicated the C-terminal conserved motif, as reported previously [9]

CsMF6p/HDM showed the highest sequence identity (82.2%) with OvMF6p/HDM, followed by FhMF6p/HDM (56.6%) and PwMF6p/HDM (50.0%) (Fig. 1). The C-terminal conserved motif, _65_LGxKIxxVxxILxxRLTxRxExY_87_ (x = any), which was reported as potential heme- and LPS-binding site [9], was conserved in all MF6p/HDM homologues. A putative signal peptide sequence was identified in the N-terminal region of CsMF6p/HDM.

### Expression of rCsMF6p/HDM and production of anti-CsMF6p/HDM

**Fig. 2:**
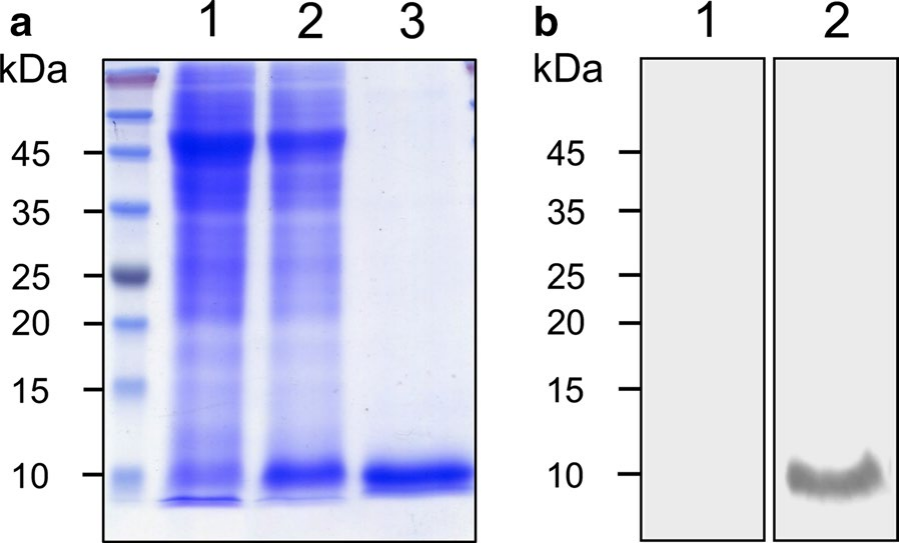
Production of recombinant CsMF6p/HDM and anti-CsMF6/HDM. **a** Expression and purification of recombinant CsMF6p/HDM. Lane 1: *E. coli* lysate control; Lane 2: IPTG-induced *E. coli* lysate; Lane 3; CsMF6p/HDM purified by Ni-NTA affinity column. **b** Validation of anti-CsMF6p/HDM. The anti-CsMF6p/HDM was collected from mice immunized with CsMF6p/HDM and confirmed by immunoblot. Lane 1: normal serum; Lane 2: anti-CsMF6p/HDM

A soluble form of rCsMF6p/HDM was expressed in *E. coli*. The approximate molecular mass of the rCsMF6p/HDM was 9 kDa, consistent with the estimated molecular mass of the deduced amino acid sequence of CsMF6p/HDM with 6 histidine residues (Fig. 2a). A polyclonal antibody against rCsMF6p/HDM (anti-CsMF6p/HDM) was successfully produced in mice immunized with rCsMF6p/HDM and the specificity of the antibody was confirmed by immunoblot (Fig. 2b).

### Expression profile of CsMF6p/HDM at the developmental stages

**Fig. 3:**
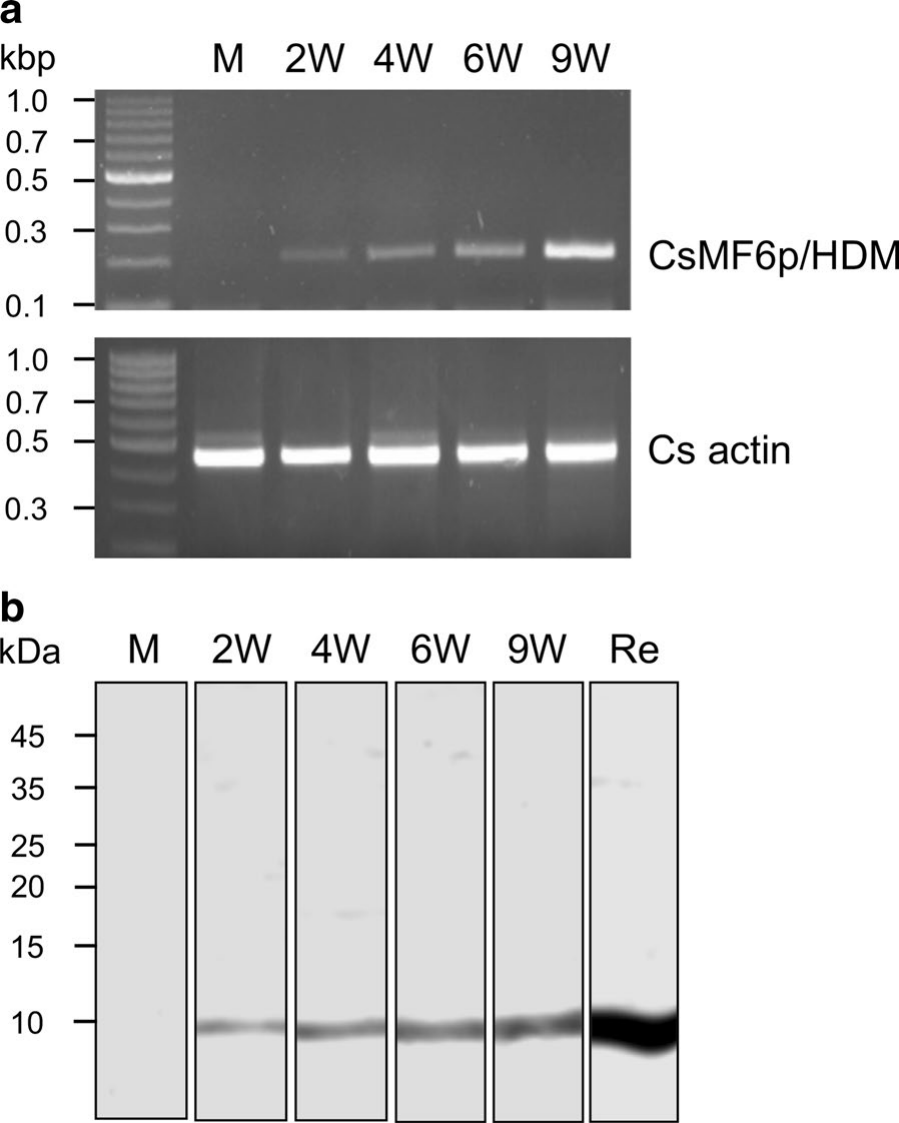
Expression pattern of CsMF6p/HDM. **a** Transcriptional profiles of CsMF6p/HDM genes at various developmental stages of *C. sinensis*. The *C. sinensis* actin gene was amplified as an internal control. **b** Immunoblot. Each developmental stage of *C. sinensis* worm extract was proved with anti-CsMF6p/HDM antibody. *Abbreviations*: M, metacercariae; 2W, 2-week-old juveniles; 4W, 4-week-old adults; 6W, 6-week-old adults; 9W, 9-week-old adult worm; Re; Recombinant CsMF6p/HDM

The CsMF6p/HDM mRNA was expressed in both juvenile and adult worms, but not in the metacercariae. The mRNA level increased gradually with the maturation of the worms from juveniles to adults (Fig. 3a). The expression of CsMF6p/HDM protein was also analyzed using immunoblot with the anti-CsMF6p/HDM and *C. sinensis* worm lysates. CsMF6p/HDM was recognized only in juvenile and adult worm extracts and its expression was increased with maturation, consistent with the mRNA expression pattern in the developmental stages (Fig. 3b).

### CsMF6p/HDM binds to LPS

**Fig. 4:**
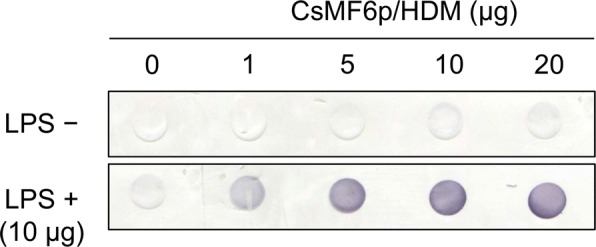
LPS-binding assay. Serial concentrations of CsMF6p/HDM (1 to 20 μg) were incubated with LPS (10 μg) coated or non-coated blot and proved with anti-CsMF6p/HDM

To evaluate LPS-binding ability of rCsMF6p/HDM, a dot blot assay was performed. The rCsMF6p/HDM did not show positive response in the absence of LPS, but showed a positive response in the presence of LPS in a dose-dependent manner, suggesting that rCsMF6p/HDM bound to LPS (Fig. 4).

### Overexpression of IL-6 and TNF-α in RAW 264.7 cells stimulated by rCsMF6p/HDM

**Fig. 5:**
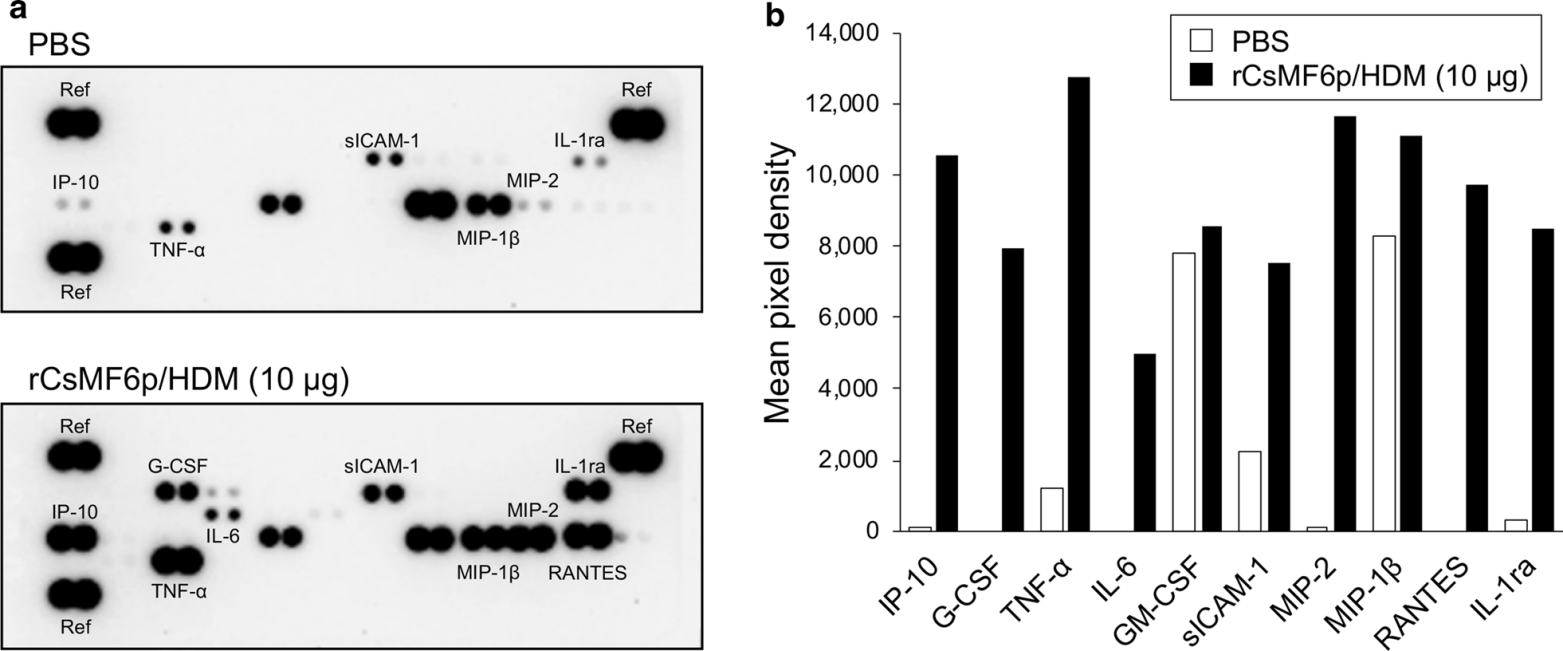
Cytokine profile analysis of RAW 264.7 cells stimulated by CsMF6p/HDM. **a** RAW 264.7 cells were stimulated with CsMF6p/HDM (10 μg/ml) for 12 h and the culture supernatants were collected and subjected to cytokine array analysis. The cells treated with PBS were used as a negative control. The results of one of two independent experiments which revealed similar expression patterns are shown. **b** Quantitative analysis of mean pixel density from the cytokine array assay. Data are representative of two independent experiments. *Abbreviations*: G-CSF, granulocyte-colony stimulating factor; IP-10, interferon gamma-induced protein 10; IL-1ra, interleukin-1 receptor antagonist; IL-6, interleukin 6; MIP-1, macrophage inflammatory protein; MIP-2, macrophage inflammatory protein 2; RANES, regulated upon activation normal T cell expressed and secreted; sICAM-1, soluble intercellular adhesion molecule-1; TNF-α, tumor necrosis factor*-*α

Expression profiles of diverse cytokines and chemokines in rCsMF6p/HDM-treated and untreated RAW 264.7 cells were comparatively analyzed. The expression of several cytokines and chemokines was increased in RAW 264.7 cells stimulated with rCsMF6p/HDM (Fig. 5a). Compared with negative control (PBS-treated), the levels of several cytokines and chemokines including interferon gamma-induced protein 10 (IP-10), macrophage inflammatory protein 2 (MIP-2), tumor necrosis factor*-*α (TNF-α), interleukin-1 receptor antagonist (IL-1ra) and interleukin 6 (IL-6) were increased in RAW 264.7 cells stimulated with rCsMF6p/HDM (Fig. 5b). These results suggest that rCsMF6p/HDM induces the synthesis of various pro-inflammatory cytokines and chemokines in RAW 264.7 cells.

### rCsMF6p/HDM does not neutralize LPS-mediated pro-inflammatory responses in RAW 264.7 cells

**Fig. 6:**
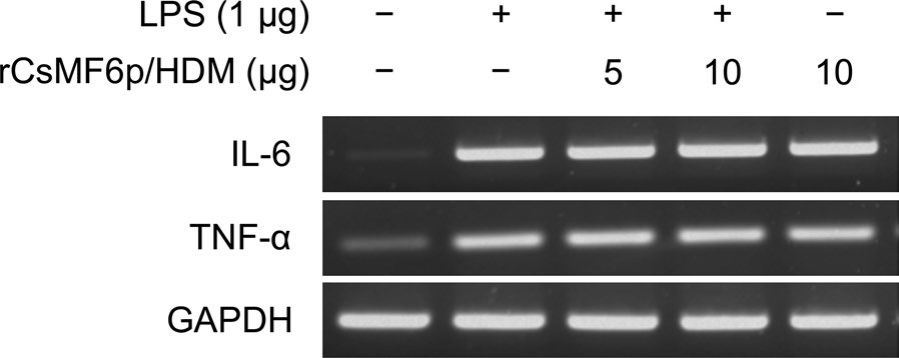
Neutralizing effect of CsMF6p/HDM in LPS-induced inflammatory response. Transcriptional activities of IL-6 and TNF-α were measured in RAW 264.7 cells, which were treated with the mixture of CsMF6p/HDM (5 or 10 μg) and LPS (1 μg)

To determine the ability of rCsMF6p/HDM to neutralize LPS-induced pro-inflammatory responses, LPS incubated with different concentrations of rCsMF6p/HDM was treated to RAW 264.7 cells and expression profiles of IL-6 and TNF-α in the cells were analyzed. Compared with LPS control, the expression of IL-6 and TNF-α in RAW 264.7 cells was not affected by rCsMF6p/HDM, indicating that rCsMF6p/HDM did not effectively neutralize the pro-inflammatory effect of LPS in RAW 264.7 cells (Fig. 6). Further, increased expression of IL-6 and TNF-α was observed in RAW 264.7 cells treated with only rCsMF6p/HDM, suggesting that CsMF6p/HDM induces pro-inflammatory responses in RAW 264.7 cells (Fig. 6).

### rCsMF6p/HDM induces IL-6 and TNF-α expression *via* MAPK signaling pathways in RAW 264.7 cells

**Fig. 7:**
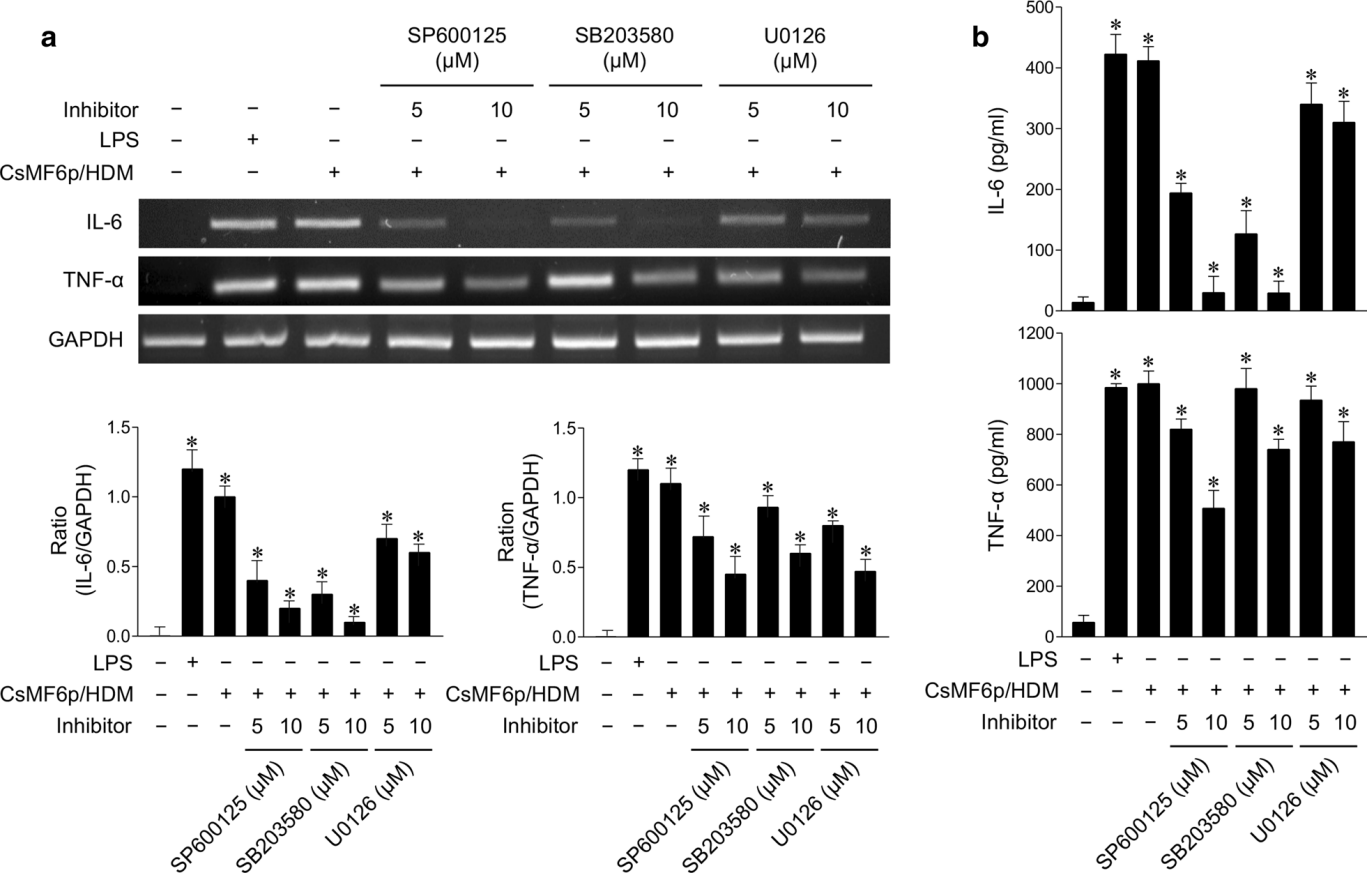
Effect of p38, JNK and ERK inhibitors on the expression of TNF-α and IL-6 in RAW 264.7 cells stimulated by CsMF6p/HDM. **a** RAW 264.7 cells were pretreated with different concentration (5 or 10 μM) of JNK inhibitor (SP600125), p38 inhibitor (SB203580), or ERK inhibitor (U0126) for 3 h before the cells were incubated with CsMF6p/HDM (10 μg) and LPS (0.5 μg) for 12 h. The cells were harvested and the expression levels of TNF-α and IL-6 were analyzed by RT-PCR. Graphs show the densitometric ratios of TNF-α and IL-6 to GAPDH. **b** ELISA analysis. Cytokine production profiles were analyzed by quantitative ELISA

To investigate the signaling pathways associated with rCsMF6p/HDM-induced IL-6 and TNF-α production, the effects of MAPKs inhibitors on rCsMF6p/HDM-mediated expression of IL-6 and TNF-α in RAW 264.7 cells were analyzed. The cells were pretreated with different concentrations (5 or 10 μM) of each inhibitor, i.e. p38 (SB203580), JNK (SP600125) or ERK (U0126), followed by stimulation with rCsMF6p/HDM. The RT-PCR analysis indicated that the inhibition of p38 and JNK impaired the ability of rCsMF6p/HDM to induce IL-6 expression in RAW 264.7 cells (Fig. 7a). Inhibition of ERK also reduced the expression of IL-6, but less than the inhibitors for p38 and JNK. Treatment with MAPK inhibitors also reduced the expression of TNF-α in RAW 264.7 cells; however, only the high inhibitor concentration (10 μM) attenuated the TNF-α expression in the cells (Fig. 7a). The effects of MAPK inhibitors on the expression of IL-6 and TNF-α were further analyzed by ELISA. The rCsMF6p/HDM-induced IL-6 and TNF-α protein expressions in RAW 264.7 cells were reduced by treatment with the inhibitors, and were consistent with RT-PCR results (Fig. 7b).

### rCsMF6p/HDM-induced IL-6 and TNF-α expression is regulated *via* NF-κB signaling pathway in RAW 264.7 cells

**Fig. 8:**
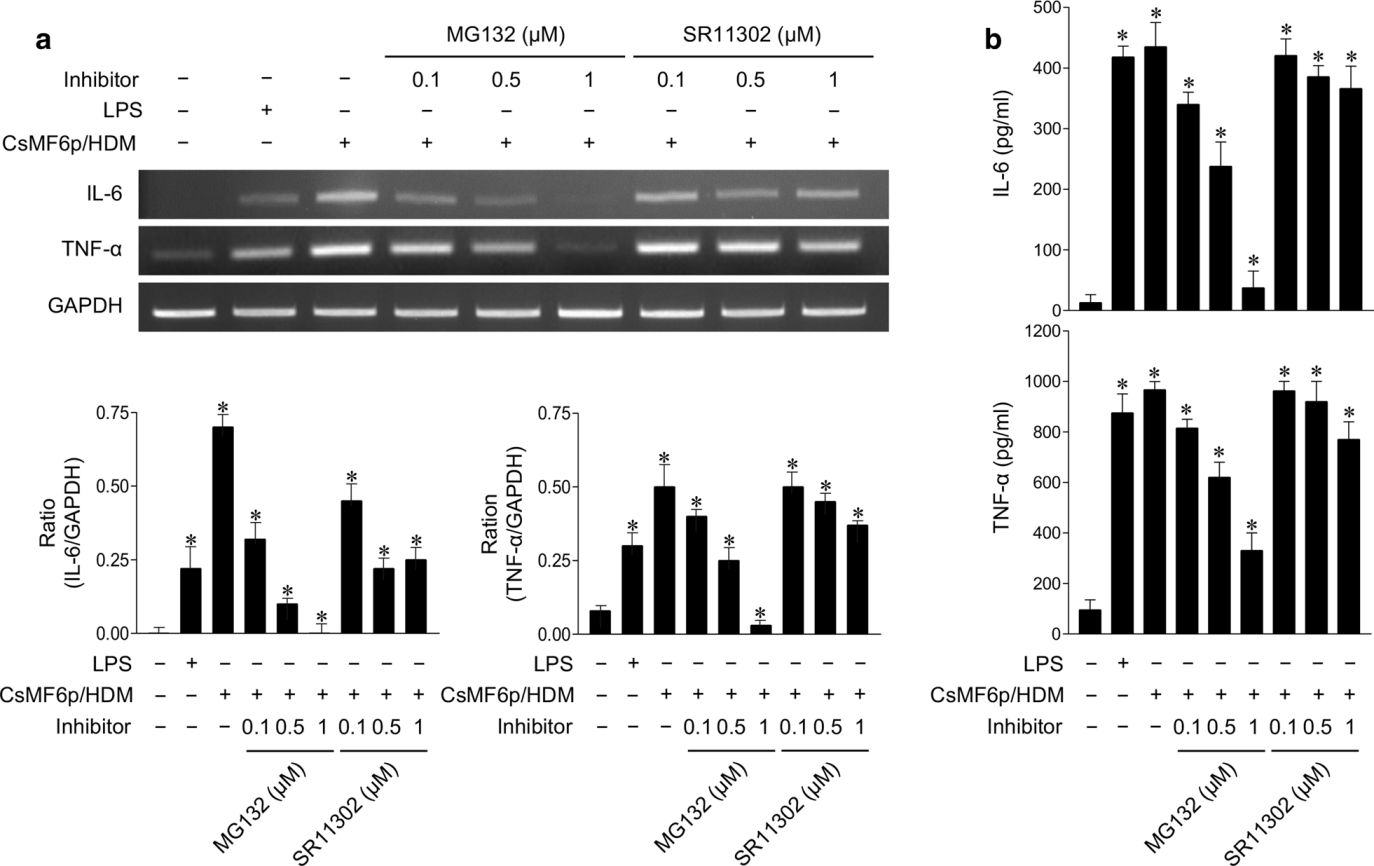
Effect of NF-κB and AP-1 inhibitors on the expression of TNF-α and IL-6 in RAW 264.7 cells stimulated with CsMF6p/HDM. **a** RAW 264.7 cells were pretreated with different concentrations (0.1, 0.5, 1.0 μM) of NF-κB inhibitor (MG132) or AP-1 inhibitor (SR11302) for 3 h, and the cells were then incubated with CsMF6p/HDM (10 μg) and LPS (0.5 μg) for 12 h. The cells were harvested and the expression levels of TNF-α and IL-6 were analyzed by RT-PCR. Graphs show the densitometric ratios of TNF-α and IL-6 to GAPDH. **b** ELISA analysis. Cytokine production profiles were analyzed by quantitative ELISA

To examine whether the enhanced expression of IL-6 and TNF-α in RAW 264.7 cells stimulated by rCsMF6p/HDM was mediated by NF-κB or AP-1 activation, the effect of NF-κB and AP-1 inhibitors on IL-6 and TNF-α expression was analyzed. The cells were pre-treated with serial concentrations (0.1, 0.5 and 1.0 μM) of inhibitors of NF-κB (MG132) or AP-1 (SR11302) and subsequently stimulated with CsMF6p/HDM. RT-PCR analysis revealed that MG132 abrogated the ability of rCsMF6p/HDM to induce IL-6 and TNF-α expression in RAW 264.7 cells in a dose-dependent manner; however, the expression of IL-6 and TNF-α was not greatly affected by SR11302 (Fig. 8a). Consistent with the RT-PCR analysis, the levels of IL-6 and TNF-α protein in rCsMF6p/HDM-stimulated RAW 264.7 cells were strongly inhibited by MG132, but not by SR11302 (Fig. 8b).

### The most appropriate structures of MF6p/HDMs within liver flukes

**Fig. 9:**
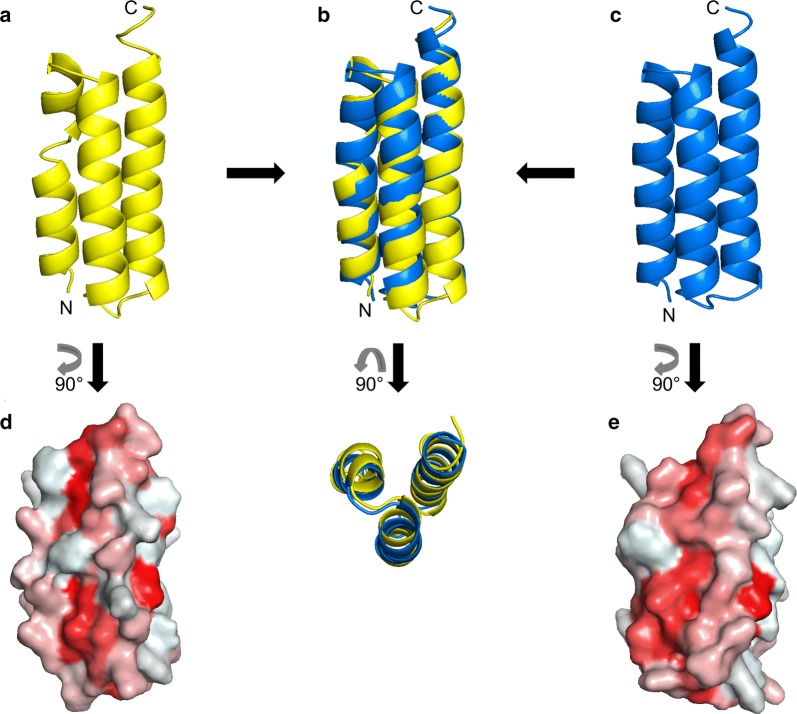
Structural comparison between CsMF6p/HDM and FhMF6/HDM. Two 3D models, CsMF6p/HDM in yellow (**a**) and FhMF6p/HDM in blue (**c**), were superposed (**b**). The superposed structure is in front view (top) and perpendicular view (bottom). Hydrophobic regions of CsMF6p/HDM (**d**) and FhMF6p/HDM (**e**) models are visualized from grey to red according to the hydrophobicity value

To elucidate the structural characteristics of trematode MF6p/HDM homologues, the top five 3D models of CsMF6p/HDM, FhMF6p/HDM, OvMF6p/HDM and PwMF6p/HDM were built and comparatively analyzed. A structural similarity dendrogram and a 20 × 20 matrix of pairwise similarities were computed for a set of 20 structures (Additional file 1: Figure S1). Among them, CsMF6p/HDM_5, FhMF6p/HDM_1, OvMF6p/HDM_5 and PwMF6p/HDM_1 were selected as the initial models according to high structural similarity based on pairwise structural comparisons across four species. All of the selected models showed the highest C-score among their individual predicted models: CsMF6p/HDM_5 (− 1.70), FhMF6p/HDM_1 (− 1.03), OvMF6p/HDM_1 (− 1.54) and PwMF6p/HDM_1 (− 1.08). The selected models were further refined and determined as the final models. For example, the final 3D model of CsMF6p/HDM was obtained by sequential refinement (Additional file 2: Figure S2, Additional file 3: Figure S3). Superposition between CsMF6p/HDM and FhMF6p/HDM yielded a TM-score of 0.83, revealing a high structural resemblance of the two molecules (Fig. 9a–c), despite varying hydrophobic surfaces (Fig. 9d, e).

### Preferable docking poses of LPS to CsMF6p/HDM and FhMF6p/HDM

To analyze the patterns of preferable LPS binding to CsMF6p/HDM and FhMF6p/HDM, 99 docking simulations and frequency analyses were performed for each binding complex. CsMF6p/HDM and FhMF6p/HDM were predicted to bind LPS with multiple and unique poses with low frequency (1–2%), resulting in a set of 99 binding poses (Additional file 4: Table S1, Additional file 5: Table S2). In the FhMF6p/HDM, a total of 25 poses out of 99 binding poses (mean binding energy of − 3.26E+04 kcal/mol) were predicted. However, no favorable binding affinity was observed for the CsMF6p/HDM. LPS was predicted to bind differently with CsMF6p/HDM and FhMF6p/HDM when all the binding residues of CsMF6p/HDM-LPS complex were normalized to those of FhMF6p/HDM-LPS complex (Additional file 6: Figure S4). An abundance of residues involved in LPS binding were predicted at the N- and C-terminal regions of CsMF6p/HDM, but in the middle and C-terminal regions of FhMF6p/HDM. When the interactions of LPS with CsMF6p/HDM and FhMF6p/HDM were optimized based on their estimated binding affinities, hydrogen bonding interactions were predicted for both proteins (Additional file 7: Figure S5). The optimized binding pose of CsMF6p/HDM with LPS suggested hydrogen bonding interactions between LPS and amino acid residues such as Arg27, Arg31, Lys36, Trp38, Lys68 and Arg79 in the N-terminal region of CsMF6p/HDM (Additional file 7: Figure S5a), but suggested unfavorable binding affinity (9.54E+04 kcal/mol) (Additional file 7: Figure S5c). The optimum binding pose of FhMF6p/HDM with LPS revealed hydrogen bonding between LPS and Arg53 of FhMF6p/HDM but other interactions (polar and hydrophobic bonds) were also predicted in the C-terminal region (Additional file 7: Figure S5b), resulting in highly favorable binding affinity (− 5.69E+04 kcal/mol) (Additional file 7: Figure S5c).

## Discussion

MF6p/HDMs are a broad family of small proteins secreted by helminth parasites [9]. Although the physiological roles of MF6p/HDMs in trematode parasites have not been clearly established, several studies suggested two interesting biological functions of this family of proteins. The molecules act as heme scavengers and transporters to maintain heme homeostasis in trematode parasites, indicating an essential role in parasite survival by detoxifying the toxic free form [9, 14]. Their ability to bind to LPS and prevent LPS-induced inflammation in mice as well as their ability to suppress antigen processing and presentation in macrophages also suggested an immunomodulatory role in suppressing host immune response, which facilitated prolonged parasite survival in the hosts [10–13, 36, 37].

Here, the biochemical and immunological properties of CsMF6p/HDM were investigated to obtain further insight into the nature of the protein. CsMF6p/HDM is a protein that shares a high level of amino acid sequence similarity with the orthologs from other trematodes. In particular, the C-terminal motif, _65_LGxKIxxVxxILxxRLTxRxExY_87_ (x = any), which has been reported as a potential heme- and LPS-binding site [9] was well conserved in CsMF6p/HDM. Expression of this protein in various developmental stages of *C. sinensis*, ranging from juveniles to adults, suggests an essential biological role in the parasitic life-cycle in mammalian hosts. FhMF6p/HDM, the most extensively studied MF6p/HDM, has attracted attention due to its immune modulatory effect against LPS-induced inflammation by preventing the activation of macrophages [10]. Although the C-terminal motif of CsMF6p/HDM showed a high sequence identity (61.8%) with that of FhMF6p/HDM, which was experimentally confirmed to bind with LPS effectively [9], the rCsMF6p/HDM did not neutralize the pro-inflammatory activity of LPS in RAW 264.7 macrophage cells. Rather, rCsMF6p/HDM induced pro-inflammatory immune responses, characterized by the release of IL-6 and TNF-α (cytokines which play key role in orchestrating the inflammation responses), in RAW 264.7 macrophage cells. The pro-inflammatory responses induced by rCsMF6p/HDM are mediated *via* the NF-κB-dependent MAPK signaling pathway.

A simple dot blot analysis using rCsMF6p/HDM and LPS revealed that LPS bound to the recombinant protein, which was not consistent with a previous study suggesting that CsMF6p/HDM may not bind to LPS effectively [9]. This disagreement may be attributed to the use of only a small fragment of the C-terminal region of CsMF6p/HDM, and not the full-length protein, in the previous study. The structural integrity of the C-terminal region in the MF6p/HDM has been considered essential for interaction with LPS [9]; however, this interaction may not be universal across similar peptides derived from MF6p/HDM orthologs in other trematodes. Otherwise, this phenomenon might be attributed to differences in the physicochemical properties of CsMF6p/HDM and FhMF6p/HDM, such as isoelectric point [9], hydrophobicity [37] and LPS serotypes (O127:B8 for CsMF6p/HDM; LPS O111:B4 and O55:B5 for FhMF6p/HDM) [38]. To understand the different patterns of CsMF6p/HDM and FhMF6p/HDM in LPS binding and neutralization of LPS-induced inflammatory responses, the docking poses of LPS with CsMF6p/HDM and FhMF6p/HDM were compared. Since the PDB template of MF6p/HDM for 3D homology modeling was unavailable in the present study, a combined method was used to build appropriate 3D models with high quality [39, 40]. A combined approach involving threading-based modeling and all-against-all comparison within the MF6p/HDM subfamily proteins was used to develop the most reliable 3D model of CsMF6p/HDM. Remarkably, although each final 3D model was selected based on the Dali Z-scores *via* pairwise structural comparison, each 3D model showed the highest C-score value. The 3D model of MF6p/HDM homologues was predicted using an *ab initio* modeling method, QUARK [41], previously [9]. Similar structures were also obtained in this study, but the models were not selected due to lower quality and similarity across different species of MF6p/HDM compared with the final structures determined in this study. Based on the final 3D models obtained, the best poses of LPS-CsMF6p/HDM binding were predicted by docking simulation analysis. The N-terminal region of CsMF6p/HDM was most probably involved in binding with LPS despite the unfavorable binding energies. Meanwhile, the C-terminal region of FhMF6p/HDM appears to be a critical region for LPS binding considering that this region showed high affinity binding energies in accordance with a previous study [9]. Moreover, the CsMF6p/HDM and FhMF6p/HDM showed different patterns of binding to LPS. These results collectively suggest that FhMF6p/HDM bind to LPS more preferably compared with CsMF6p/HDM, which was consistent with previous results [9]. Low-affinity binding between CsMF6p/HDM and LPS may result in insufficient capture of LPS by rCsMF6p/HDM. Therefore, the rCsMF6p/HDM failed to effectively neutralize LPS-induced inflammatory response in RAW 264.7 cells.

Interestingly, the rCsMF6p/HDM induced pro-inflammatory responses, which was mediated *via* NF-κB-dependent MAPK signaling pathway. The pro-inflammatory immune responses in diverse types of mammalian cells induced by C. *sinensis* excretory and secretory products (ESP) and proteins were reported previously [42–45]. CsMF6p/HDM is a protein largely identified in the ESP of *C. sinensis*, which indicates that the protein is actively released from the parasite and continuously interacts with the biliary epithelium in bile ducts. Considering that pro-inflammatory immune responses are induced in RAW 246.7 macrophage cells by rCsMF6p/HDM, this protein may mediate continuous inflammatory response in bile epithelial cells and may be associated with inflammatory events in clonorchiasis.

## Conclusions

CsMF6p/HDM is a protein belonging to the MF6p/HDM protein family found in trematode parasites. The expression pattern of CsMF6p/HDM in different developmental stages of *C. sinensis* suggests its key role in parasite physiology and interactions with host. Although rCsMF6p/HDM binds to LPS, the binding affinity may not be sufficient to maintain a stable complex of rCsMF6p/HDM and LPS. Therefore, rCsMF6p/HDM failed to neutralize LPS-induced inflammatory response in RAW 246.7 cells. Moreover, the rCsMF6p/HDM-induced pro-inflammatory response is characterized by the release of IL-6 and TNF-α in RAW 264.7 macrophage cells. The pro-inflammatory response induced by rCsMF6p/HDM is mediated *via* NF-κB-dependent MAPK signaling pathway. These results collectively suggest that CsMF6p/HDM mediates *C. sinensis*-induced inflammation cascades that eventually lead to hepatobiliary diseases.

## Supplementary information

**Additional file 1: Figure S1.** All-against-all comparison of CsMF6p/HDM and homologues.

**Additional file 2: Figure S2.** Quality verification of initial 3D model of CsMF6p/HDM.

**Additional file 3: Figure S3.** Quality verification of final 3D model of CsMF6p/HDM.

**Additional file 4: Table S1.** Docking results of CsMF6p/HDM and LPS (99 simulations).

**Additional file 5: Table S2.** Docking results of FhMF6p/HDM and LPS (99 simulations).

**Additional file 6: Figure S4.** Difference of binding residues of LPS to between CsMF6p/HDM and FhMF6p/HDM.

**Additional file 7: Figure S5.** Interactions of LPS between CsMF6p/HDM and FhMF6p/HDM.

## Data Availability

The data supporting the conclusions of this article are provided within the article and its additional files. The original datasets analyzed in the present study are available from the corresponding author upon request.

## Abbreviations

Anti-CsMF6p/HDM: polyclonal antibody against CsMF6p/HDM
AP-1: activator protein-1
CsMF6p/HDM: MF6p/HDM of *Clonorchis sinensis*
ELISA: enzyme-linked immunosorbent assay
ERK: extracellular signal-regulated kinase
ESP: excretory and secretory products
G-CSF: granulocyte-colony stimulating factor
GAPDH: glyceraldehyde 3-phosphate dehydrogenase
HDM: MF6p/host defense molecule
IL-1ra: interleukin-1 receptor antagonist
IL-6: interleukin-6
IP-10: interferon gamma-induced protein 10
IPTG: isopropyl-1-thio-β-d-galactopyranoside
I-TASSER: Iterative Threading ASSEmbly Refinement
JNK: c-Jun N-terminal kinase
LPS: lipopolysaccharide
MAPKs: mitogen-activated protein kinases
MIP-2: macrophage inflammatory protein 2
NF-κB: nuclear factor-kappa B
Ni-NTA: nickel-nitrilotriacetic acid
PBS: phosphate-buffered saline
RANES: regulated upon activation normal T cell expressed and secreted
sICAM-1: soluble intercellular adhesion molecule-1
TNF-α: tumor necrosis factor-α
3D: three-dimensional
