# Supplementary figures and images for: Clonorchis sinensis MF6p/HDM (CsMF6p/HDM) induces pro-inflammatory immune response in RAW 264.7 macrophage cells via NF-κB-dependent MAPK pathways
Source: Parasit Vectors. 2020 Jan 13;13:20. doi: 10.1186/s13071-020-3882-0 (PMC6958574; doi:10.1186/s13071-020-3882-0)

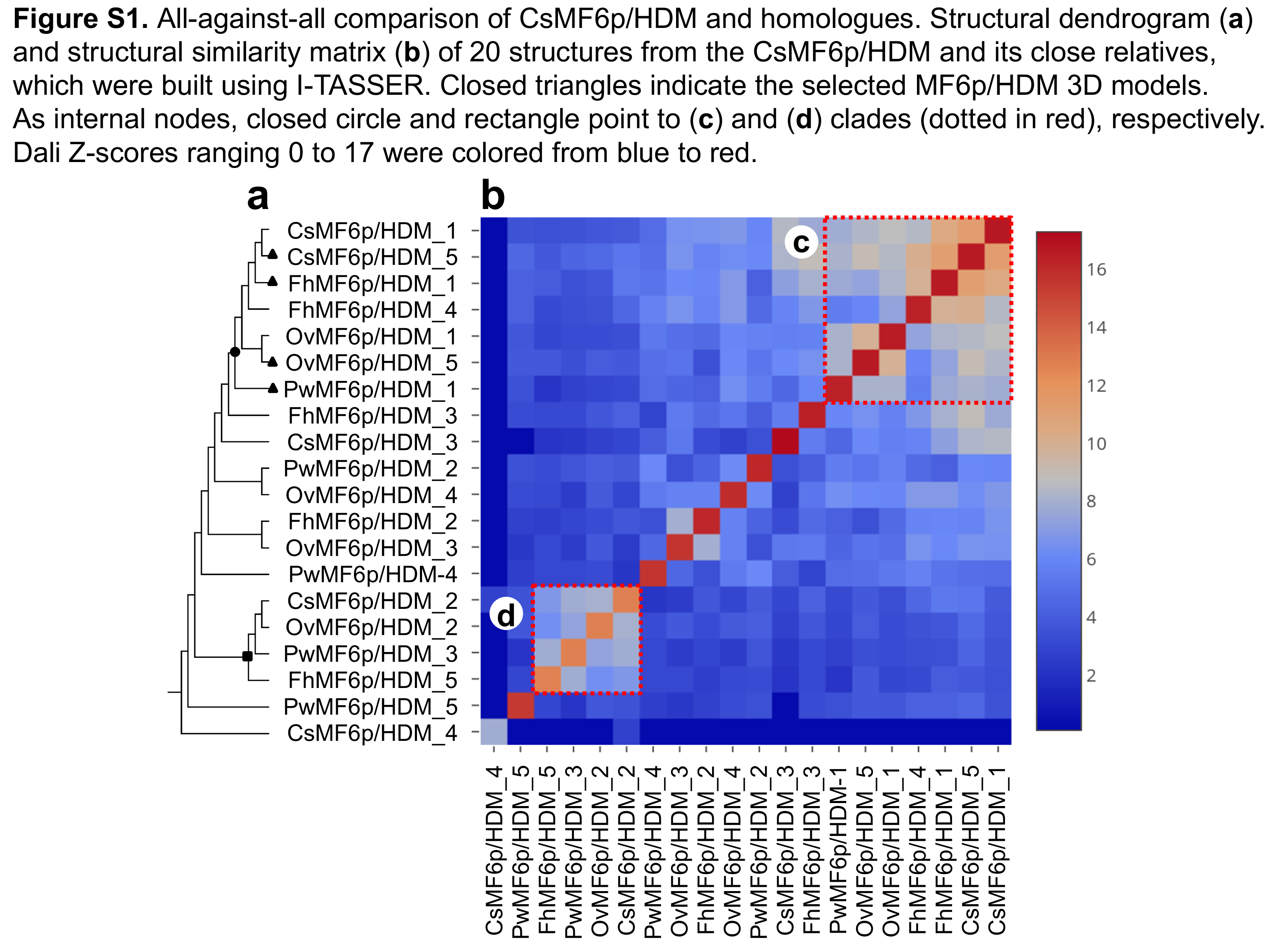

Supplement: Supplementary file 1 — Additional file 1: Figure S1. All-against-all comparison of CsMF6p/HDM and homologues. [file 13071_2020_3882_MOESM1_ESM.tif]

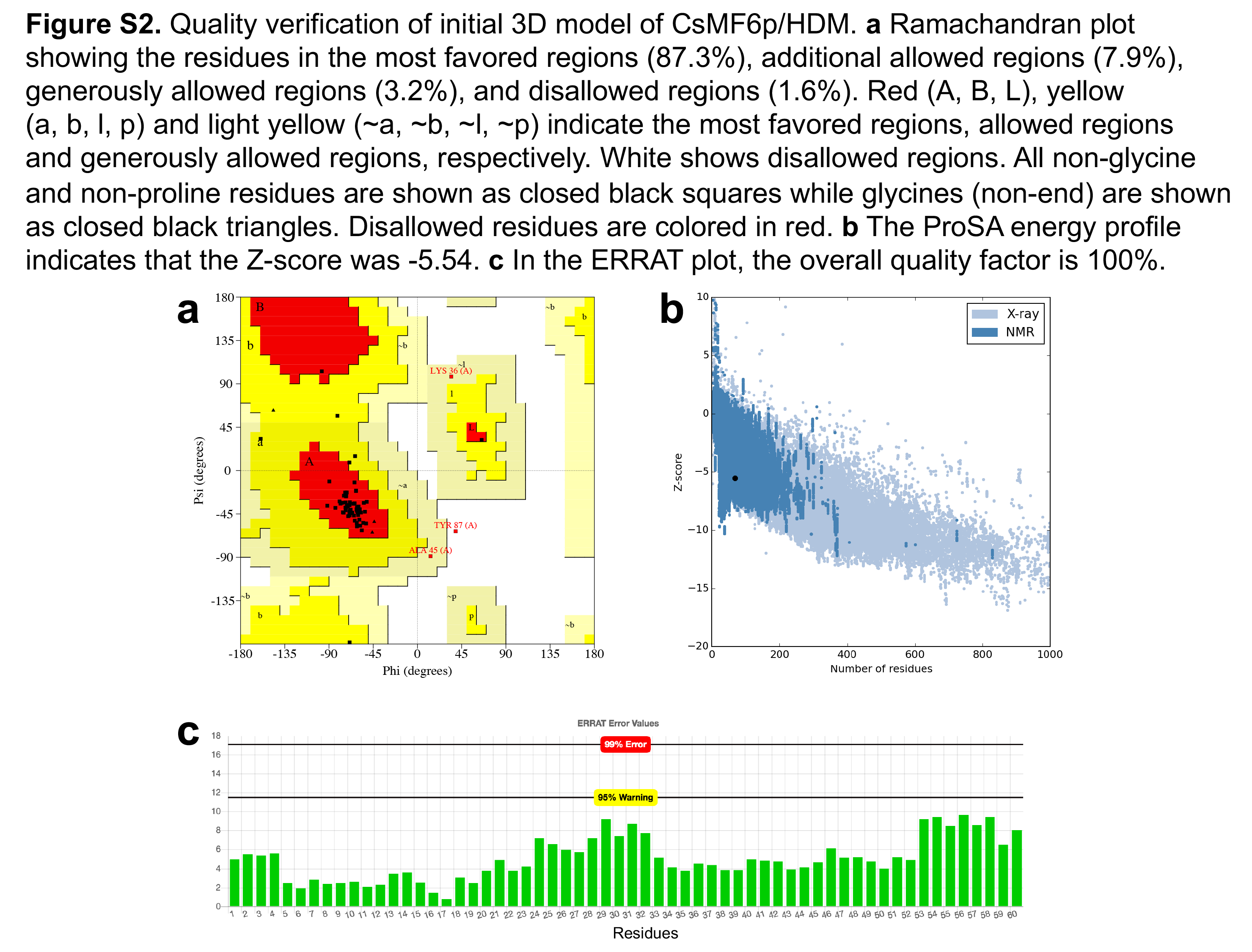

Supplement: Supplementary file 2 — Additional file 2: Figure S2. Quality verification of initial 3D model of CsMF6p/HDM. [file 13071_2020_3882_MOESM2_ESM.tif]

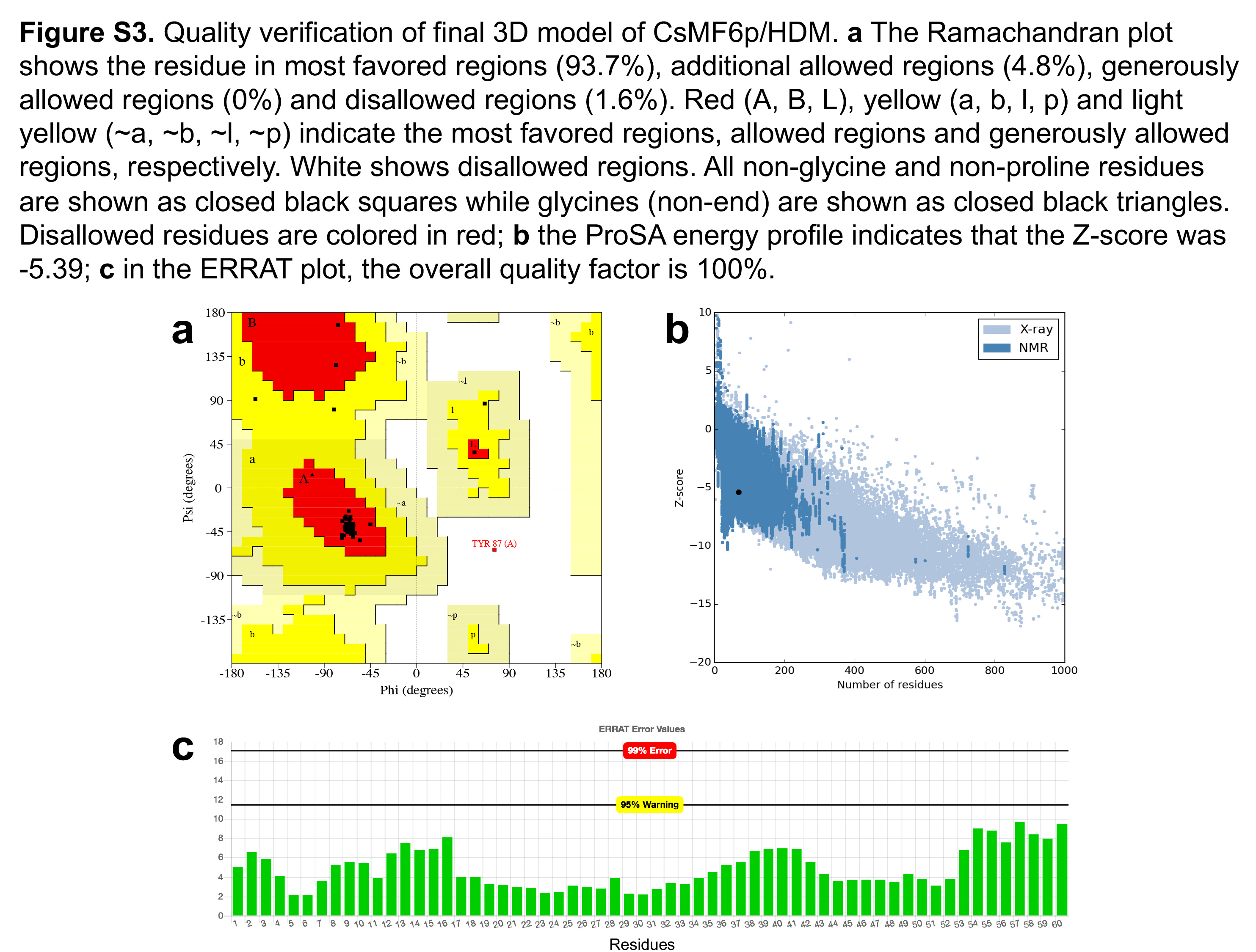

Supplement: Supplementary file 3 — Additional file 3: Figure S3. Quality verification of final 3D model of CsMF6p/HDM. [file 13071_2020_3882_MOESM3_ESM.tif]

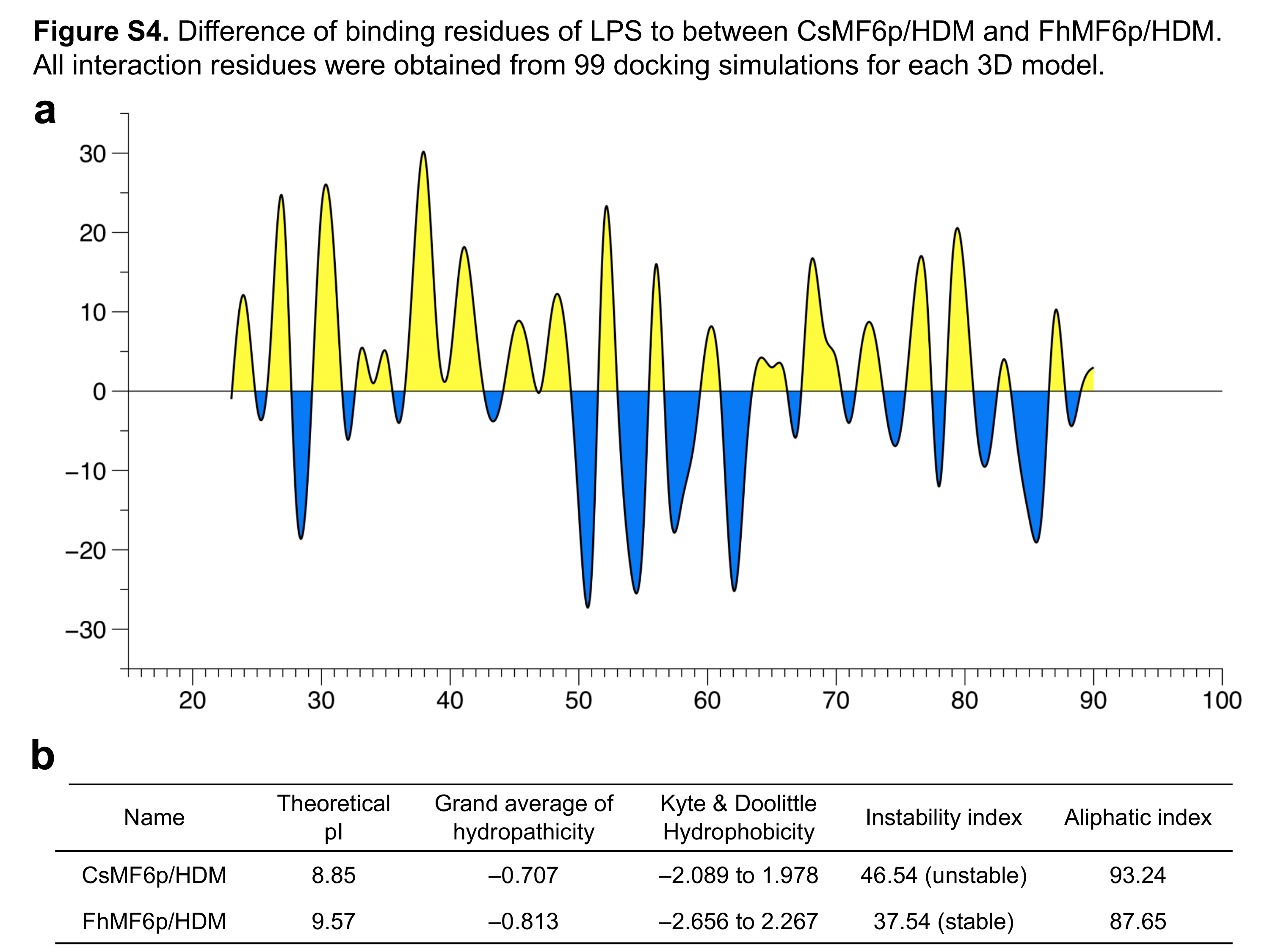

Supplement: Supplementary file 6 — Additional file 6: Figure S4. Difference of binding residues of LPS to between CsMF6p/HDM and FhMF6p/HDM. [file 13071_2020_3882_MOESM6_ESM.tif]

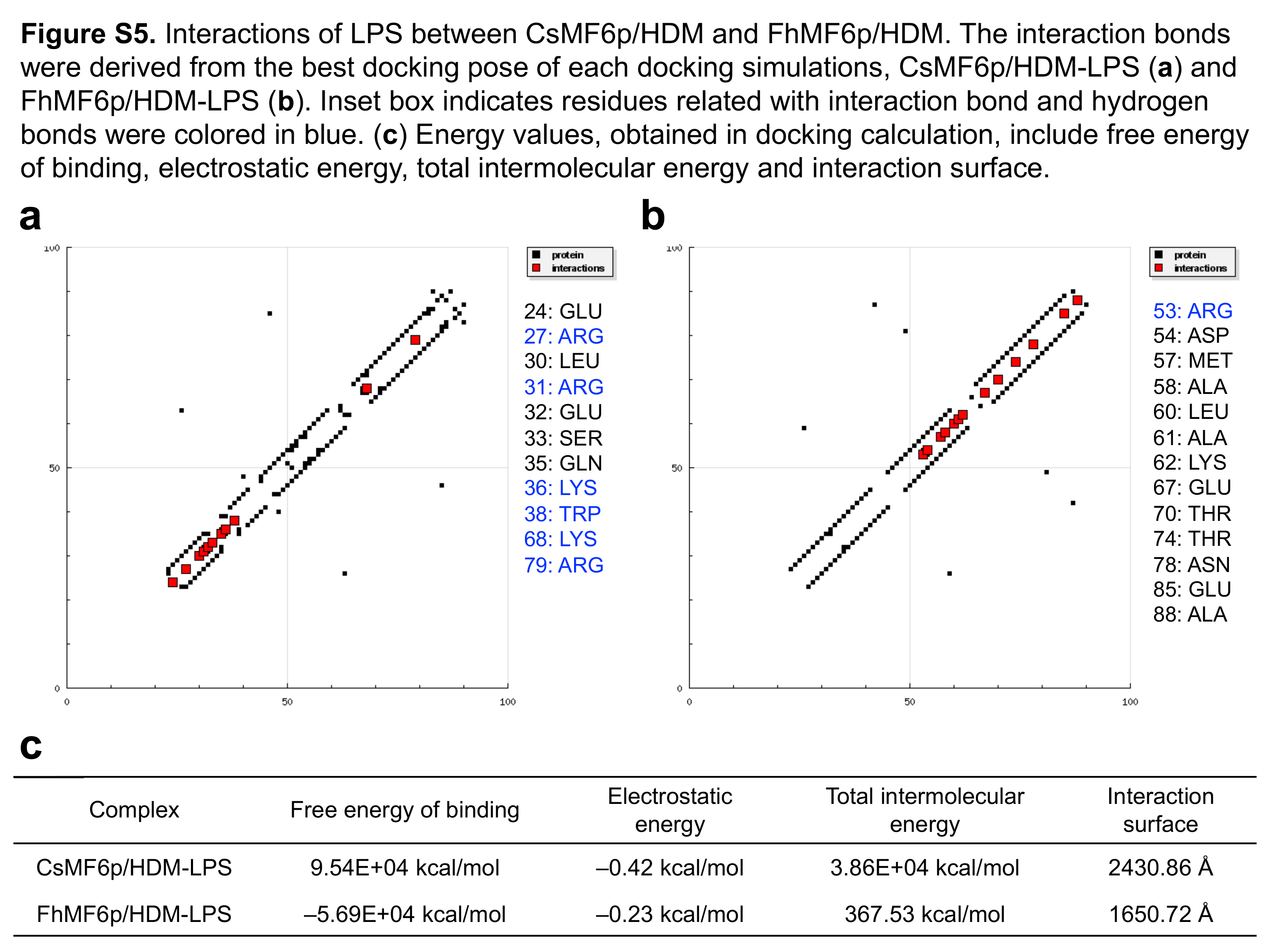

Supplement: Supplementary file 7 — Additional file 7: Figure S5. Interactions of LPS between CsMF6p/HDM and FhMF6p/HDM. [file 13071_2020_3882_MOESM7_ESM.tif]
