# Supplementary material for: Clonorchis sinensis MF6p/HDM (CsMF6p/HDM) induces pro-inflammatory immune response in RAW 264.7 macrophage cells via NF-κB-dependent MAPK pathways
Source: Parasit Vectors. 2020 Jan 13;13:20. doi: 10.1186/s13071-020-3882-0 (PMC6958574; doi:10.1186/s13071-020-3882-0)
Supplement: Supplementary file 4 — Additional file 4: Table S1. Docking results of CsMF6p/HDM and LPS (99 simulations). [file 13071_2020_3882_MOESM4_ESM.pdf]

Additional file 4: Table S1. Docking results of CsMF6p and LPS (100 simulations)

Rank	Est. Free Energy of Binding	vdW + Hbond + desolv Energy	Electrostatic Energy	Total Intermolec. Energy	Frequency	Interact. Surface
1	9.54E+04	3.86E+04	-0.42	3.86E+04	1%	2430.86
2	9.93E+04	8.93E+04	-0.35	8.93E+04	1%	2173.93
3	1.45E+05	2.33E+04	0.5	2.33E+04	1%	1591.55
4	1.47E+05	9.64E+03	-0.34	9.64E+03	1%	1833.46
5	1.58E+05	4.83E+04	0.14	4.83E+04	1%	2431.65
6	1.77E+05	2.05E+05	-0.4	2.05E+05	1%	1983.63
7	1.85E+05	6.30E+04	0.79	6.30E+04	1%	2272.82
8	2.12E+05	1.05E+05	1.7	1.05E+05	1%	1810.15
9	2.56E+05	2.67E+05	-0.2	2.67E+05	1%	1652.61
10	2.62E+05	2.26E+05	0	2.26E+05	2%	2140.50
11	2.65E+05	1.35E+05	1.2	1.35E+05	1%	1674.80
12	2.90E+05	1.40E+05	1.6	1.40E+05	1%	2187.02
13	3.14E+05	5.67E+04	-0.8	5.67E+04	1%	1967.09
14	3.25E+05	3.27E+05	-2.6	3.27E+05	1%	2108.05
15	3.44E+05	1.54E+05	-0.38	1.54E+05	1%	2159.09
16	3.88E+05	2.30E+05	0.07	2.30E+05	1%	1879.04
17	3.89E+05	3.33E+05	-1.18	3.33E+05	1%	2230.51
18	4.01E+05	1.05E+05	-2.39	1.05E+05	1%	1706.46
19	4.12E+05	2.30E+05	-0.36	2.30E+05	1%	2054.11
20	4.21E+05	3.78E+05	0.56	3.78E+05	1%	1985.87
21	4.61E+05	5.13E+05	0.62	5.13E+05	1%	2279.33
22	4.63E+05	4.60E+05	1.21	4.60E+05	1%	2471.36
23	4.72E+05	3.77E+05	0.81	3.77E+05	1%	2105.18
24	4.96E+05	4.68E+05	0.23	4.68E+05	1%	2541.83
25	4.96E+05	4.04E+05	-0.35	4.04E+05	1%	1969.92
26	4.96E+05	2.80E+05	-0.26	2.80E+05	1%	1891.35
27	5.07E+05	2.62E+05	-0.81	2.62E+05	1%	2316.71
28	5.24E+05	2.85E+05	-1.28	2.85E+05	1%	2023.64
29	5.27E+05	2.01E+05	-0.5	2.01E+05	1%	2410.78
30	5.48E+05	5.98E+05	1.46	5.98E+05	1%	2235.66
31	5.64E+05	3.17E+05	1.21	3.17E+05	1%	1637.43
32	5.69E+05	4.45E+05	-0.51	4.45E+05	1%	2008.42
33	5.83E+05	4.24E+05	3.09	4.24E+05	1%	2019.77
34	6.06E+05	6.11E+05	-0.91	6.11E+05	1%	2233.35
35	6.12E+05	4.48E+05	-1.13	4.48E+05	1%	2129.58
36	6.64E+05	6.44E+05	-0.64	6.44E+05	1%	2201.02
37	6.88E+05	5.82E+05	0.38	5.82E+05	1%	2160.52
38	7.10E+05	7.43E+05	0.29	7.43E+05	1%	2265.14
39	7.35E+05	6.14E+05	-1.11	6.14E+05	1%	2263.38
40	7.46E+05	6.28E+05	-0.35	6.28E+05	1%	1857.27
41	7.49E+05	7.40E+05	-0.27	7.40E+05	1%	2406.75
42	7.53E+05	6.69E+05	0.32	6.69E+05	1%	2286.29
43	8.15E+05	6.67E+05	-1.24	6.67E+05	1%	1704.40
44	8.44E+05	3.09E+05	-0.21	3.09E+05	1%	1997.19
45	8.50E+05	7.11E+05	-5.43	7.11E+05	1%	2229.84
46	8.65E+05	7.93E+05	0.7	7.93E+05	1%	2178.54
47	8.84E+05	5.29E+05	-0.27	5.29E+05	1%	2067.62
48	8.93E+05	8.33E+05	-0.74	8.33E+05	1%	2220.39
49	8.97E+05	5.56E+05	0.62	5.56E+05	1%	2286.54
50	9.05E+05	3.82E+05	-2.28	3.82E+05	1%	1951.40
51	9.22E+05	8.41E+05	-0.78	8.41E+05	1%	2335.35
52	9.42E+05	8.43E+05	1.54	8.43E+05	1%	2299.53
53	9.73E+05	5.52E+05	0.12	5.52E+05	1%	2304.82
54	9.90E+05	6.82E+05	-2.99	6.82E+05	1%	2089.44
55	9.92E+05	9.16E+05	-0.66	9.16E+05	1%	2130.57
56	9.94E+05	8.77E+05	-1.09	8.77E+05	1%	2213.57
57	9.98E+05	7.35E+05	1.18E+05	8.53E+05	1%	1892.00
58	1.01E+06	6.16E+05	-0.72	6.16E+05	1%	1873.41
59	1.02E+06	6.26E+05	-0.11	6.26E+05	1%	2053.99
60	1.02E+06	8.54E+05	-0.77	8.54E+05	1%	2281.22
61	1.03E+06	1.04E+06	-1.81	1.04E+06	1%	1860.62
62	1.06E+06	9.48E+05	0.82	9.48E+05	1%	1981.84
63	1.06E+06	8.14E+05	0.28	8.14E+05	1%	2172.53
64	1.07E+06	8.75E+05	-2.54	8.75E+05	1%	1922.26
65	1.07E+06	7.98E+05	-1.84	7.98E+05	1%	2232.29
66	1.07E+06	9.55E+05	1.18	9.55E+05	1%	2306.19
67	1.07E+06	7.79E+05	-0.13	7.79E+05	1%	1865.98
68	1.08E+06	6.46E+05	0.09	6.46E+05	1%	2188.72
69	1.08E+06	9.00E+05	-2.54	9.00E+05	1%	2368.48
70	1.08E+06	1.08E+06	-0.16	1.08E+06	1%	2210.82
71	1.09E+06	9.92E+05	0.41	9.92E+05	1%	1866.25
72	1.11E+06	9.66E+05	-0.51	9.66E+05	1%	2114.03
73	1.15E+06	1.00E+06	0.33	1.00E+06	1%	2138.44
74	1.15E+06	1.12E+06	-1.57	1.12E+06	1%	2020.57
75	1.16E+06	9.98E+05	-0.33	9.98E+05	1%	2501.00
76	1.18E+06	8.32E+05	0.99	8.32E+05	1%	2605.99
77	1.21E+06	1.15E+06	-0.47	1.15E+06	1%	2156.45
78	1.25E+06	8.96E+05	-0.41	8.96E+05	1%	2366.40
79	1.29E+06	3.54E+05	1.15	3.54E+05	1%	1975.07
80	1.31E+06	8.99E+05	-0.51	8.99E+05	1%	2186.24

81	1.32E+06	1.04E+06	0.44	1.04E+06	1%	2162.02
82	1.35E+06	7.78E+05	1.08E+05	8.86E+05	1%	1934.61
83	1.36E+06	1.10E+06	-1.02	1.10E+06	1%	2348.34
84	1.39E+06	1.08E+06	0.78	1.08E+06	1%	2230.15
85	1.40E+06	1.16E+06	-4.18	1.16E+06	1%	2140.30
86	1.40E+06	1.22E+06	-0.03	1.22E+06	1%	2047.32
87	1.42E+06	1.41E+06	-1.23	1.41E+06	1%	2450.43
88	1.42E+06	1.34E+06	4.53	1.34E+06	1%	2350.86
89	1.43E+06	1.38E+06	-1	1.38E+06	1%	2288.03
90	1.48E+06	1.36E+06	-0.89	1.36E+06	1%	2233.19
91	1.52E+06	1.03E+06	3.25	1.03E+06	1%	1961.81
92	1.60E+06	1.49E+06	1.77	1.49E+06	1%	2260.63
93	1.64E+06	1.29E+06	-0.89	1.29E+06	1%	2133.15
94	1.66E+06	1.39E+06	-0.81	1.39E+06	1%	1923.79
95	1.71E+06	9.90E+05	2.67	9.90E+05	1%	2283.73
96	1.76E+06	1.42E+06	-0.7	1.42E+06	1%	2103.30
97	1.83E+06	1.33E+06	2.97E+05	1.63E+06	1%	2115.56
98	1.85E+06	1.58E+06	-0.67	1.58E+06	1%	2263.24
99	1.90E+06	1.44E+06	0.63	1.44E+06	1%	2297.47

*Favorable binding energies were indicated in red.

*vdW: van der Waals

*Hbond: hydrogen bond

*desolv: desolvation energy
