# Supplementary material for: Clonorchis sinensis MF6p/HDM (CsMF6p/HDM) induces pro-inflammatory immune response in RAW 264.7 macrophage cells via NF-κB-dependent MAPK pathways
Source: Parasit Vectors. 2020 Jan 13;13:20. doi: 10.1186/s13071-020-3882-0 (PMC6958574; doi:10.1186/s13071-020-3882-0)
Supplement: Supplementary file 5 — Additional file 5: Table S2. Docking results of FhMF6p/HDM and LPS (99 simulations). [file 13071_2020_3882_MOESM5_ESM.pdf]

Additional file 5: Table S2. Docking results of FhMF6p and LPS (100 simulations)

Rank	Est. Free Energy of Binding	vdW + Hbond + desolv Energy	Electrostatic Energy	Total Intermolec. Energy	Frequency	Interact. Surface
1	-5.69E+04	367.76	-0.23	367.53	1%	1650.72
2	-5.48E+04	1.60E+03	-0.08	1.60E+03	1%	1495.86
3	-4.94E+04	647.23	-0.25	646.98	1%	1606.26
4	-4.93E+04	3.46E+03	0.09	3.46E+03	1%	1189.71
5	-4.63E+04	1.96E+03	0.34	1.96E+03	1%	1797.36
6	-4.61E+04	1.57E+03	0.36	1.57E+03	1%	1316.12
7	-4.59E+04	80.16	-0.24	79.92	1%	1185.34
8	-4.33E+04	7.27E+03	0.03	7.27E+03	1%	1463.93
9	-4.30E+04	2.22E+03	-0.89	2.22E+03	1%	1833.91
10	-3.37E+04	1.62E+03	-1.31	1.62E+03	1%	1738.44
11	-3.28E+04	1.25E+03	-0.27	1.25E+03	1%	1696.09
12	-3.14E+04	592.81	-0.2	592.62	1%	1346.46
13	-3.13E+04	1.53E+04	0.08	1.53E+04	1%	1579.95
14	-2.97E+04	7.84E+03	-0.89	7.84E+03	1%	1493.48
15	-2.89E+04	1.20E+03	-0.07	1.20E+03	2%	1788.77
16	-2.80E+04	142.23	-0.34	141.89	1%	1190.97
17	-2.78E+04	5.06E+03	0.22	5.06E+03	1%	1511.95
18	-2.43E+04	7.41E+03	-0.72	7.41E+03	1%	1833.91
19	-2.08E+04	7.25E+03	0.01	7.25E+03	1%	1667.86
20	-2.06E+04	1.05E+04	-0.29	1.05E+04	1%	1535.66
21	-2.04E+04	1.70E+04	0.07	1.70E+04	1%	1338.17
22	-2.00E+04	1.94E+04	-0.8	1.94E+04	1%	1755.93
23	-1.71E+04	573.53	-0.18	573.36	1%	1796.53
24	-1.10E+04	2.99E+03	-0.29	2.99E+03	1%	1499.36
25	-2.36E+03	1.44E+04	-0.15	1.44E+04	1%	1385.57
26	3.53E+03	1.20E+04	-0.33	1.20E+04	1%	1416.87
27	7.07E+03	9.22E+03	0.27	9.22E+03	1%	1543.81
28	1.23E+04	2.32E+04	-0.18	2.32E+04	1%	1663.13
29	1.41E+04	9.96E+03	-1.12	9.96E+03	1%	1966.40
30	2.65E+04	1.92E+04	-0.75	1.92E+04	1%	1744.19
31	2.95E+04	1.08E+04	-0.39	1.08E+04	1%	1940.33
32	3.01E+04	1.55E+04	-0.03	1.55E+04	1%	1522.30
33	4.07E+04	818.18	0.05	818.23	1%	1604.42
34	4.37E+04	7.31E+04	-0.22	7.31E+04	1%	1171.84
35	5.47E+04	6.76E+04	0.51	6.76E+04	1%	1786.49
36	5.51E+04	2.61E+03	0.28	2.61E+03	1%	1566.75
37	5.52E+04	1.40E+03	0.25	1.40E+03	1%	1557.73
38	5.85E+04	3.28E+03	-0.24	3.28E+03	1%	1721.51
39	5.93E+04	6.97E+04	0.23	6.97E+04	1%	2144.93
40	6.09E+04	3.76E+03	-0.25	3.76E+03	1%	1700.89
41	6.78E+04	5.35E+04	-0.05	5.35E+04	1%	1897.87
42	7.15E+04	2.65E+03	0.02	2.65E+03	1%	1525.01
43	7.27E+04	1.69E+04	-0.33	1.69E+04	1%	1492.36
44	7.48E+04	7.70E+03	-0.74	7.70E+03	1%	2161.83
45	8.52E+04	6.43E+04	0.14	6.43E+04	1%	1779.11
46	8.58E+04	3.82E+03	0.23	3.83E+03	1%	1442.45
47	8.95E+04	4.84E+04	-0.21	4.84E+04	1%	1991.23
48	9.07E+04	6.73E+04	1.27	6.73E+04	1%	2252.03
49	9.42E+04	4.51E+04	-0.32	4.51E+04	1%	1935.53
50	1.09E+05	1.30E+05	-0.16	1.30E+05	1%	1765.84
51	1.14E+05	1.51E+05	-4.81	1.51E+05	1%	1900.21
52	1.29E+05	5.49E+04	-1.09	5.49E+04	1%	1953.29
53	1.46E+05	3.92E+04	0.16	3.92E+04	1%	1266.30
54	1.55E+05	1.83E+04	-0.82	1.83E+04	1%	1963.69
55	1.61E+05	1.45E+04	-0.14	1.45E+04	1%	1521.38
56	1.64E+05	1.82E+04	1.99	1.82E+04	1%	1350.30
57	1.66E+05	4.05E+04	-0.88	4.05E+04	1%	1442.24
58	1.72E+05	2.00E+05	-0.77	2.00E+05	1%	2289.56
59	1.73E+05	1.35E+05	1.38	1.35E+05	1%	1993.23
60	1.93E+05	7.05E+04	-1.01	7.05E+04	1%	1824.05
61	1.97E+05	2.22E+05	0.13	2.22E+05	1%	2039.09
62	2.05E+05	2.40E+05	-0.88	2.40E+05	1%	1618.99
63	2.07E+05	2.37E+05	-1.44	2.37E+05	1%	1754.86
64	2.12E+05	1.60E+05	0.83	1.60E+05	1%	2175.49
65	2.23E+05	5.47E+04	-0.74	5.47E+04	1%	1811.16
66	2.28E+05	6.45E+04	-0.33	6.45E+04	1%	1386.67
67	2.45E+05	2.50E+05	-1.04	2.50E+05	1%	1862.65
68	2.47E+05	9.14E+04	-0.3	9.14E+04	1%	1806.92
69	2.47E+05	4.59E+04	-0.12	4.59E+04	1%	1762.27
70	2.72E+05	2.46E+05	0	2.46E+05	1%	2061.91
71	2.76E+05	1.19E+05	-0.6	1.19E+05	1%	1893.58
72	2.93E+05	2.06E+05	1.04	2.06E+05	1%	1955.17
73	2.94E+05	2.29E+05	1.55	2.29E+05	1%	2087.08
74	2.95E+05	9.08E+04	3	9.08E+04	1%	1578.30
75	3.07E+05	2.89E+05	0.64	2.89E+05	1%	1866.66
76	3.25E+05	5.57E+04	-0.14	5.57E+04	1%	1790.53
77	3.30E+05	2.58E+05	0.24	2.58E+05	1%	1891.40
78	3.38E+05	1.45E+05	-0.75	1.45E+05	1%	1781.34
79	4.00E+05	2.28E+05	0.18	2.28E+05	1%	1903.59
80	4.21E+05	4.24E+05	0.39	4.24E+05	1%	1643.21

81	4.44E+05	2.99E+05	3	2.99E+05	1%	1894.85
82	4.56E+05	3.47E+05	0.33	3.47E+05	1%	1731.32
83	4.92E+05	2.48E+05	-0.9	2.48E+05	1%	1800.99
84	5.08E+05	2.20E+05	1.07E+05	3.27E+05	1%	1473.93
85	5.38E+05	3.71E+05	-0.02	3.71E+05	1%	1865.41
86	5.41E+05	4.52E+05	-1.84	4.52E+05	1%	1708.42
87	5.43E+05	3.31E+05	0.92	3.31E+05	1%	1924.78
88	5.90E+05	1.80E+05	-0.61	1.80E+05	1%	1527.86
89	6.10E+05	5.75E+05	-0.61	5.75E+05	1%	1956.06
90	6.15E+05	4.70E+05	-0.53	4.70E+05	1%	1638.61
91	6.37E+05	5.16E+05	-2.23	5.16E+05	1%	2128.49
92	7.66E+05	5.96E+05	1.09	5.96E+05	1%	1527.91
93	7.76E+05	8.01E+05	-1.2	8.01E+05	1%	1976.26
94	7.86E+05	6.17E+05	3.18	6.17E+05	1%	2046.05
95	8.58E+05	7.01E+05	3.23	7.01E+05	1%	2015.06
96	9.21E+05	7.81E+05	-1.15	7.81E+05	1%	1640.43
97	9.47E+05	7.75E+05	5.4	7.75E+05	1%	2164.60
98	1.01E+06	8.29E+05	-1.65	8.29E+05	1%	2288.33
99	1.30E+06	1.19E+06	0.77	1.19E+06	1%	2097.11

*Favorable binding energies were indicated in red.

*vdW: van der Waals

*Hbond: hydrogen bond

*desolv: desolvation energy
